# HCG11 up-regulation induced by ELK4 suppressed proliferation in vestibular schwannoma by targeting miR-620/ELK4

**DOI:** 10.1186/s12935-020-01691-0

**Published:** 2021-01-05

**Authors:** Ruiqing Long, Zhuohui Liu, Jinghui Li, Yuan Zhang, Hualin Yu

**Affiliations:** 1 Otolaryngology Department, The First Affiliated Hospital of Kunming Medical University, Kunming, 650032 Yunnan China; 2 Neurosurgery Department, The First Affiliated Hospital of Kunming Medical University, No. 1 Building, No. 295 Xichang Road, Kunming, 650032 Yunnan China

**Keywords:** HCG11, miR-620, ELK4, Vestibular schwannoma

## Abstract

**Background:**

Vestibular schwannoma (VS) is a kind of benign tumor deriving from the acoustic nerve sheath. Substantial long non-coding RNAs (lncRNAs) were illustrated to have crucial roles in multiple cancers. However, few lncRNAs were elucidated in VS.

**Methods:**

HCG11, miR-620 and ELK4 expression were tested by RT-qPCR. Gain-of-function experiments were conducted to confirm the effect of HCG11 on VS.

**Results:**

HCG11 possessed a low expression in VS cell lines. Overexpression of HCG11 repressed cell proliferation but accelerated apoptosis of VS cells. Moreover, we identified ELK4 stimulated the transcription of HCG11 and their affinity was verified by ChIP assays. MiR-620 was chosen to be a target of HCG11 and it was tested to have a high expression in VS cell lines. Moreover, depletion of miR-620 could inhibit cell proliferative ability while fostering apoptosis rate of VS cells. ELK4 was low expressed in VS cell lines and knockdown of ELK4 could rescue the effects made by HCG11 overexpression on progression of VS.

**Conclusions:**

HCG11 could inhibit the growth of VS by targeting miR-620/ELK4 in VS cells. HCG11 was a novel therapeutic target for VS treatment.

## Background

Acoustic neuroma has another name called vestibular schwannoma (VS). It is a common sighted intracranial tumor but luckily it is benign and has a slow growing pace [1]. But it would become larger and repress the brain and lead to hearing loss, tinnitus and balance disorders as time goes by [2]. VS could threaten life if not treated. The current methods include surgical treatment and stereotactic radiotherapy. It will be helpful to cure this disease if the detailed molecular mechanism is understood.

Growing studies indicated that lncRNAs were active regulators in the initiation and development of diverse kinds of cancers [3, 4]. The aberrant expression of lncRNAs could function as tumor inhibitors or oncogenes [5, 6]. HCG11 was analyzed in hepatocellular carcinoma and was reported as a tumor suppressor [7]. However, the function of HCG11 was not clear in VS.

Mounting essays supported competing endogenous RNAs (ceRNAs) regulatory system could have tremendous effects on the progression of cancers. In this system, lncRNAs acted as sponges of microRNAs (miRNAs) so that their downstream targets could escape from the combination with miRNAs and coded into proteins [8, 9]. HCG11 was described to suppress the course of glioma by targeting miR-496/CPEB3 [10]. Nevertheless, regulatory system concerning HCG11 has not been applied and explained in VS till now. In our study, we focused on the role of HCG11 in VS cells.

MiRNAs were introduced to have vital functions in modulating the process of cancers [11–13]. MiR-1 was said to repress cell proliferation in VS by regulating VEGFA [14]. MiR-21 up-regulation was discovered to foster VS proliferation [15]. MiR-620 was analyzed in cervical cancer as an oncogene [16]. However, has the relationship between miR-620 and VS has never been established at all.

ELK4 was reported to exert critical functions in large numbers of articles [17, 18]. But it has not been studied in VS ever. In this study, we found it as a transcription factor of HCG11 in VS serving as the target of miR-620.

The purpose of our study was to explore the role of HCG11 in VS progression. The rescue assays illustrated the effectiveness of HCG11/miR-620/ELK4 axis in vestibular schwannoma. The axis was displayed as Additional file 1: Figure S1.

## Methods

### Tissue collection and cell culture

Informed consents had acquired from all participants in this clinical study, and protocol had been approved by the Human Studies Committee of the First Affiliated Hospital of Kunming Medical University. The freshly-harvested human tissues of control great auricular nerve (GAN; n = 50) and sporadic VS (n = 50) were collected from indicated surgeries and kept in saline solution, following transporting to laboratory on ice.

### Cell culture of normal schwanns cells (SCs) and VSs

Tissues of control and VS group in sterile PBS were cut to 1 mm^3^ pieces in DMEM/F12 medium, 1% penicillin/streptomycin (Pen/Strep), 10% fetal bovine serum (FBS) and 1% GlutaMAX (all, Thermo Fisher Scientific, Inc., Waltham, MA). To acquire purer SC population, epineurium was removed from nerve tissue via tugging and removing the outer layers under dissecting microscope. Tissues were centrifuged at 8 °C at 3000*g* for 3 min, then the tissue pellets were cultured in new medium adding 5% Collagenase (Sigma Aldrich, St. Louis, MI) and 0.5% Hyaluronidase (Sigma-Aldrich) at 37 °C for 24 h. Human VS cell line HEI-193 was derived from patients with neurofibromatosis type 2 (NF2) and used in this study.

### Real-time quantitative PCR (RT-qPCR)

TRIzol reagent (Thermo Fisher Scientific) was employed for extracting total RNA samples from cells. RNA was converted into cDNA using Reverse Transcription Kit (Toyobo, Osaka, Japan). SYBR Green Super Mix (Bio-Rad, Hercules, CA) was applied for qPCR. Relative gene expression was standardized to GAPDH or U6, and fold-changes were determined by 2^−ΔΔCT^ method.

### Cell transfection

VSs and HEI-193 cells in 24-well plates were reaped for 48 h of transfection with Lipofectamine 2000 (Invitrogen, Carlsbad, CA). The pcDNA 3.1/HCG11, pcDNA3.1/ELK4 and relative negative control (NC; termed pcDNA3.1), shRNAs of ELK4 and control (sh-NC), as well as the miR-620 mimics/inhibitor and NC mimics/inhibitor, all were procured from RiboBio (Guangzhou, China).

### Cell proliferation assays

Cells after transfection were collected and planted to the 96-well plates adding 10 μL of CCK-8 (Beyotime, Shanghai, China). Absorbance at 450 nm was tested by microplate reader to determine cell viability. Besides, the cultured cells were treated with 5-Bromo-20-Deoxyuridine (EdU; Invitrogen) for 20 h and fixed, in the presence of anti-EdU antibody (AbD Serotec, Munich, Germany).

### Cell apoptosis assays

The transfected VS cells were collected and fixed on ice for 1 h. Then they were stained by Annexin V-APC and 7-AAD, followed by detection with flow cytometry using FACSCalibur (BD Biosciences, San Jose, CA). Besides, terminal deoxynucleotidyl transferase dUTP nick end labeling (TUNEL; Roche, Basel, Switzerland) was applied to assess cell apoptosis for 1 h at 37 °C after fixation and permeabilization.

### Dual-luciferase reporter assays

For promoter analysis, HCG11 promoter covering the wild-type (WT) or mutant (Mut) ELK4 binding sites was severally inserted into luciferase reporter vector pGL3 (Promega, Madison, WI), then co-transfected with pcDNA3.1/ELK4 or pcDNA3.1 into VS cells. Besides, the pmirGLO vectors (Promega) containing the WT and Mut interacting sites of miR-620 in HCG11 sequence or ELK4 3′-UTR, were constructed and transfected with indicated plasmids into cells for 48 h. Dual-Luciferase Reporter Assay System (Promega) was used to test luciferase intensity.

### UCSC and JASPAR

UCSC Genome Browser is a website that contains sketched and annotated genomes of various species, including humans, mice and rats, and provides a range of web analytics tools. Thus, we used UCSC to find out the transcription factors of HCG11. In addition, the JASPAR database (http://jaspar.genereg.net/) offers open data access collected from publications or experimentally defined results for those who are looking for models for specific transcription factor binding sites. In this study, JASPAR was utilized to predict binding sites of transcription factors on the gene promoters.

### ChIP assay

The crosslinked chromatin was first cut into 200–1000-bp fragments for the immunoprecipitation with 2 μg of anti-ELK4 or negative control anti-IgG antibody (Millipore, Billerica, MA), along with the 30 μL of magnetic beads. Precipitated chromatin DNA after purification was subjected to RT-qPCR.

### FISH

RNA FISH probe for HCG11 was synthesized by Ribobio for incubation with VS cells in hybridization solution. After nuclear counterstaining with DAPI, cells were photographed under fluorescence microscope (Leica, Wetzlar, Germany).

### RNA pull-down

The protein extracts from cultured cells were collected and cultivated with the biotinylated miR-620 (Bio-miR-620-Wt/Mut) and Bio-NC. The RNA–protein mixtures collected by beads were analyzed by RT-qPCR.

### RIP

EZ-Magna RIP RNA Binding Protein Immunoprecipitation Kit (Millipore) was utilized for RIP assay in VS cells, in the presence of anti-Argonaute 2 (Ago2) antibody (Millipore) or negative control anti-IgG (Millipore). After isolating the precipitant RNAs, RT-qPCR was followed.

### Statistical analyses

Data were all statistically analyzed by t test or one-way ANOVA with the help of SPSS version 19.0 (SPSS Inc., Chicago, IL), with the significant level at p-value < 0.05. Results from 3 or more repeats were displayed as the mean ± standard deviation (SD).

## Results

### HCG11 up-regulation suppressed the proliferation and fostered apoptosis of VS cells

**Fig. 1:**
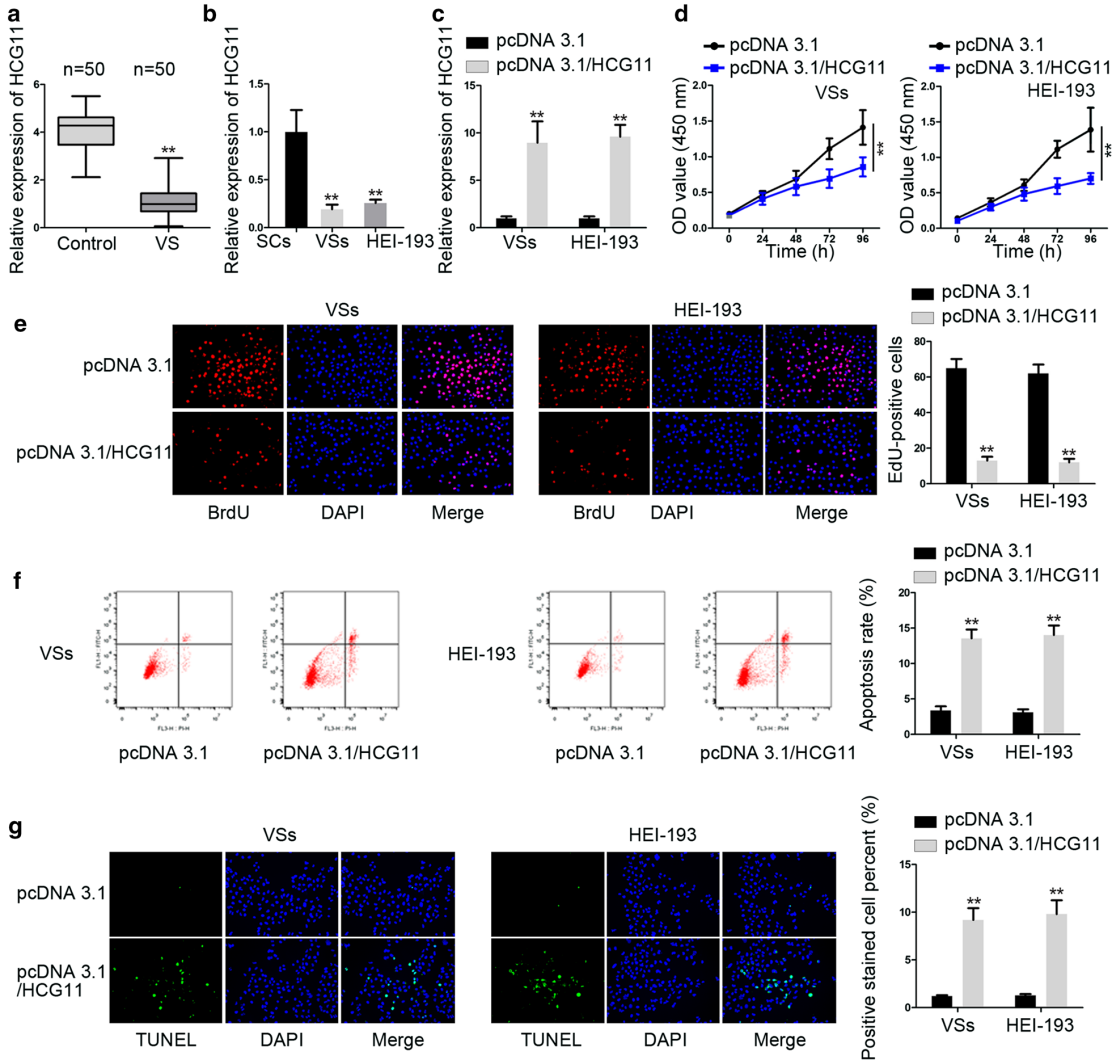
HCG11 up-regulation suppressed the proliferation and fostered apoptosis of VS cells. **a** HCG expression was tested in VS tissue and normal SCs tissue. **b** HCG11 expression was detected in VSs and HEl-193 cells and normal SCs cells. **c** The expression of HCG11 was examined in cells receiving transfection of pcDNA3.1 and pcDNA3.1/HCG11. **d**, **e** CCK8 and EdU assays were applied to measure cell proliferation abilities in cells transfected with pcDNA3.1 and pcDNA3.1/HCG11. **f**, **g** Apoptosis rate was determined in cells undergoing transfection with pcDNA3.1 and pcDNA3.1/HCG11. *P < 0.05, **P < 0.01

To have a clear understanding of HCG11 in VS, first we tested the expression of HCG11 in VS tissue. The outcomes of RT-qPCR revealed that the expression of HCG11 was low in VS tissue (Fig. 1a). Then, we detected the expression of HCG11 in VSs and HEl-193 and normal schwanns (SCs) cells. HEl-193 cell was collected from VS patients. Data of RT-qPCR showed that HCG11 harbored a remarkably low expression in VS cell lines compared with normal SCs cells (Fig. 1b). PcDNA3.1/HCG11 was transfected into cells and the results manifested that HCG11 expression was increased by pcDNA3.1/HCG11 (Fig. 1c). The outcome of CCK8 and EdU assays showcased that cell proliferation ability was decreased sharply by HCG overexpression in comparison with negative control (Fig. 1d, e). Apoptosis rate examined by flow cytometry analysis and TUNEL assays were enhanced significantly in comparison with negative control (Fig. 1f, g). In short, HCG11 was low-expressed in VS tissues and cells. And overexpression of HCG11 could inhibit proliferation but promoted apoptosis of VS cells.

### ELK4 stimulated transcription of HCG11 in VS cells

**Fig. 2:**
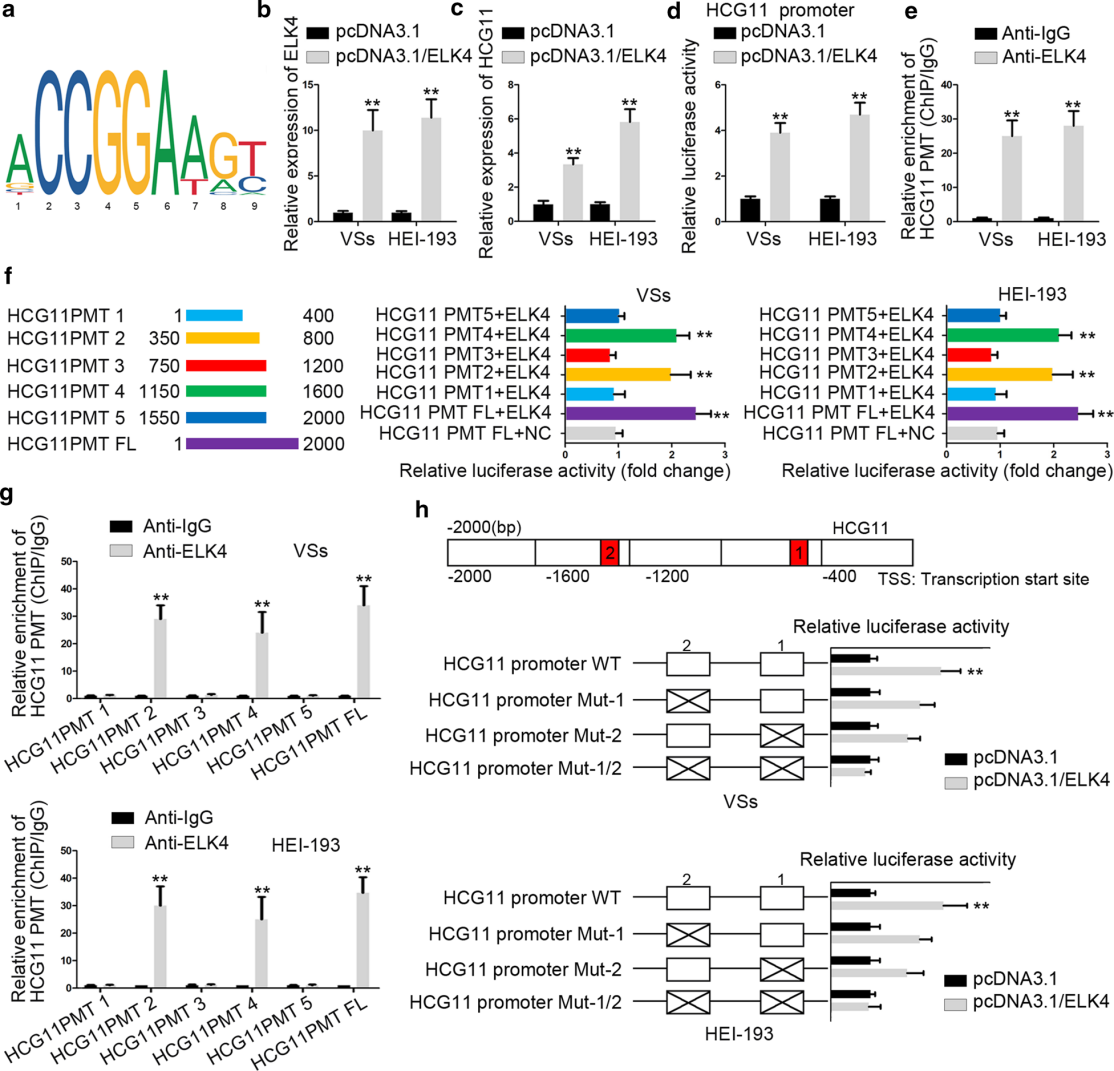
ELK4 stimulated transcription of HCG11 in VS cells. **a** The motif structure was projected by bioinformatics. **b** RT-qPCR was built to determine the expression of ELK4 in cells treated with pcDNA3.1/ELK4. **c** HCG11 expression was evaluated in cells treated with pcDNA3.1/ELK4. **d** Luciferase reporter assays were carried out to verify the combination between ELK4 and HCG11. **e** ChIP assays were established to certify the affinity between ELK4 and HCG11. **f** Luciferase reporter assays were performed to testify the relationship between ELK4 and HCG11 PMT. **g** ChIP assays were used to confirm the interaction between ELK4 and HCG11 PMT. **h** Luciferase reporter assays were constructed to find out the binding place between ELK4 and HCG11 PMT. *P < 0.05, **P < 0.01

Then, we searched UCSC website (http://genome.uscs.edu.) to find out the transcription factors of HCG11. ELK4 was introduced to have activating transcription function and the motif structure of ELK4 was shown in Fig. 2a from JASPAR (http://jaspar.genereg.net/). The expression of ELK4 was examined by RT-qPCR after adding pcDNA3.1/ELK4. The outcomes disclosed that ELK4 expression was increased (Fig. 2b). To analyze the effect of ELK4 on HCG11, pcDNA3.1/ELK4 was transfected into cells. RT-qPCR data manifested that HCG11 expression was increased distinctly compared with negative control (Fig. 2c). The consequences of luciferase reporter assays displayed that pcDNA3.1/ELK4 could elevate the activity overtly (Fig. 2d). Moreover, results of ChIP experiments exhibited that HCG11 promoters (PMT) were enriched in ELK4 not IgG (Fig. 2e). These results illustrated the interplay between ELK4 and HCG11. To find out the exact position of binding sites, we divided HCG11 PMT binding sites into 6 pieces. The cells received co-transfection with each piece and ELK4. The results of luciferase reporter assays presented that full length of HCG11 PMT, HCG11 PMT 2 (350–800), HCG11 PMT 4 (1150–1600) could enhance the luciferase activity prominently (Fig. 2f). The data from ChIP assays delineated that full length of HCG11, HCG11 PMT 2&4 had enrichment in ELK4 (Fig. 2g). Therefore, we inferred that there were binding sites in 400–800 domain and 1200–1600 domain. These two parts were marked with 1 and 2. Next, we conducted luciferase reporter assays and the outcomes portrayed that relative luciferase activity was enhanced conspicuously via transfecting pcDNA3.1/ELK4 and HCG11 PMT WT compared with negative control group. The activities in cells with HCG11 PMT Mut 1 or Mut 2 were weaker than that with HCG11 PMT WT. However, no evident changes could be observed in the plasmid with HCG11 PMT Mut-1/2 (Fig. 2h). In brief, ELK4 could accelerate transcription of HCG11 in VS cells.

### MiR-620 down-regulation suppressed the growth of VS

**Fig. 3:**
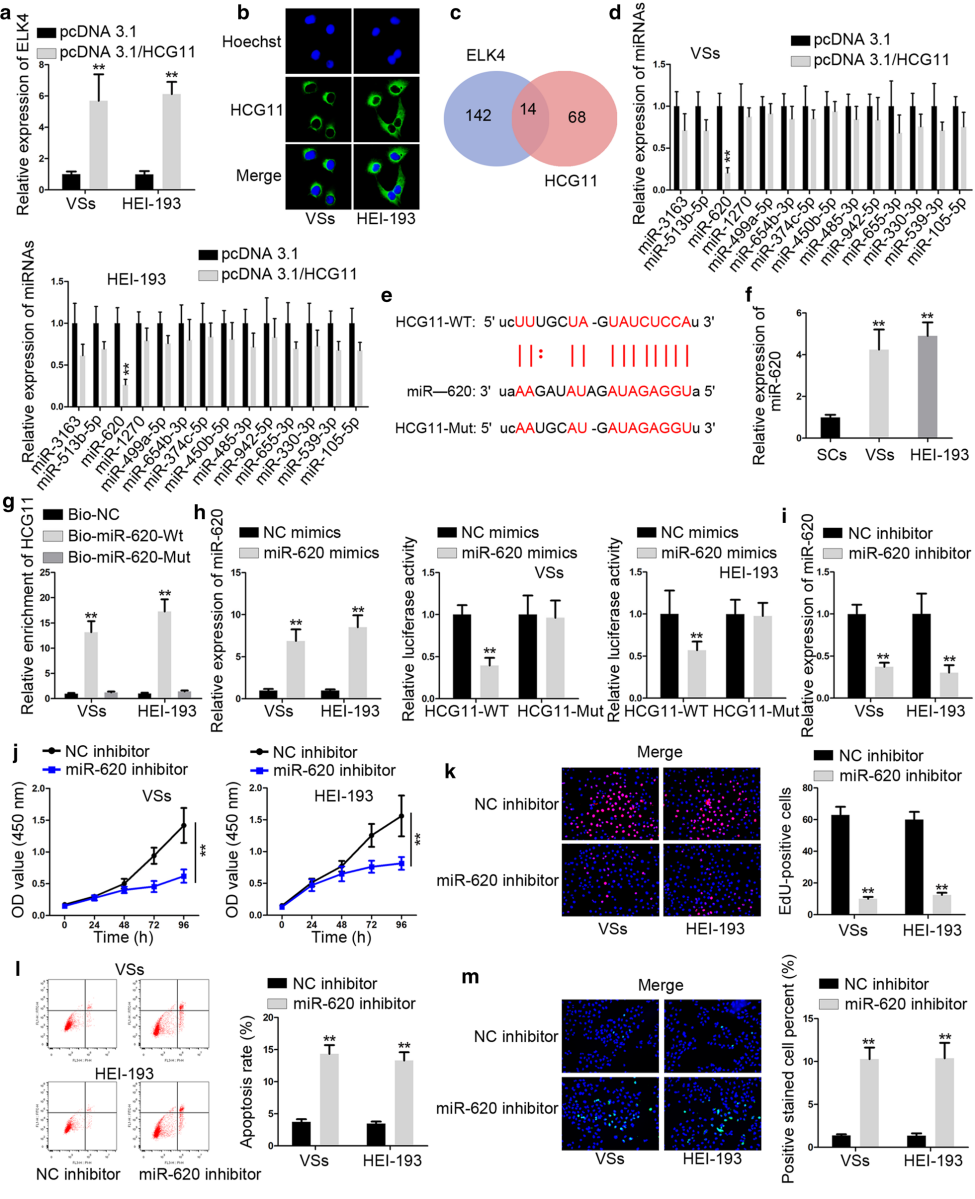
MiR-620 down-regulation suppressed the growth of VS. **a** ELK4 expression was examined by RT-qPCR in cells transfected with pcDNA3.1/HCG11. **b** FISH experiments were built to judge the place of HCG11 in VS cells. **c** Venn diagram showed miRNAs could both bind to ELK4 and HCG11. **d** 14 miRNAs expressions were examined in cells transfected with pcDNA3.1/HCG11. **e** Bioinformatics projected the binding sequences between HCG11 and ELK4. **f** MiR-620 expression was appraised in VS cells. **g** RNA pull down was constructed to confirm the relationship between HCG11 and miR-620. **h** RT-qPCR was used to test the expression of miR-620 and luciferase reporter assays were performed to verify the combination between miR-620 and HCG11. **i** RT-qPCR was used to assess the expression of miR-620 in cells treated with miR-620 inhibitor. **j**, **k** CCK8 and EdU assays were built to probe the cell proliferative capacities in cells transfected with pcDNA3.1/HCG11 and miR-620 inhibitor. **l**, **m** The apoptosis rate was estimated in flow cytometry analysis and TUNEL in cells received transfection with pcDNA3.1/HCG11 and miR-620 inhibitor. *P < 0.05, **P < 0.01

Then, pcDNA3.1/HCG11 was transfected into cells to confirm the effect of HCG11 on ELK4 expression. Results from RT-qPCR displayed that ELK4 expression was elevated by pcDNA3.1/HCG11 (Fig. 3a). To explore the role of HCG11 in ceRNA system, first, we applied FISH experiments and the outcomes presented that HCG11 amassed in cytoplasm (Fig. 3b). Then, we used bioinformatics to find that miRNAs had links both with HCG11 and ELK4. As shown in Fig. 3c, there were 14 miRNAs could bind to both HCG11 and ELK4. PcDNA3.1/ELK4 was transfected into cells and results from RT-qPCR showed that only miR-620 expression was declined dramatically by pcDNA3.1/ELK4 (Fig. 3d). The binding sequences were projected by bioinformatics (Fig. 3e). Next, we detected the expression of miR-620 in VS cells and the consequences revealed that miR-620 expression was up-regulated in VS cells (Fig. 3f). The outcomes of RNA pulldown assays indicated that miR-620 could bind to HCG11 (Fig. 3g). MiR-620 mimics were transfected into cells to elevate the expression of miR-620. The data of luciferase reporter assays showed that miR-620 mimics could reduce the activity of plasmid with HCG11-WT but had no effect on plasmid with HCG11-Mut (Fig. 3h). MiR-620 inhibitor was transfected into cells and the expression of miR-620 was diminished by miR-620 inhibitor (Fig. 3i). Then, we studied the effect of miR-620 on VS progression. The consequences of CCK8 and EdU assays suggested that miR-620 depletion weakened cell proliferation ability (Fig. 3j, k). The apoptosis examined by flow cytometry analysis and TUNEL assays was increased by miR-620 down-regulation (Fig. 3l, m). In conclusion, miR-620 could bind to HCG11 and miR-620 depletion could inhibit proliferative capacity while enhance apoptosis rate of VS cells.

### HCG11 repressed the process of VS by up-regulating ELK4 expression

**Fig. 4:**
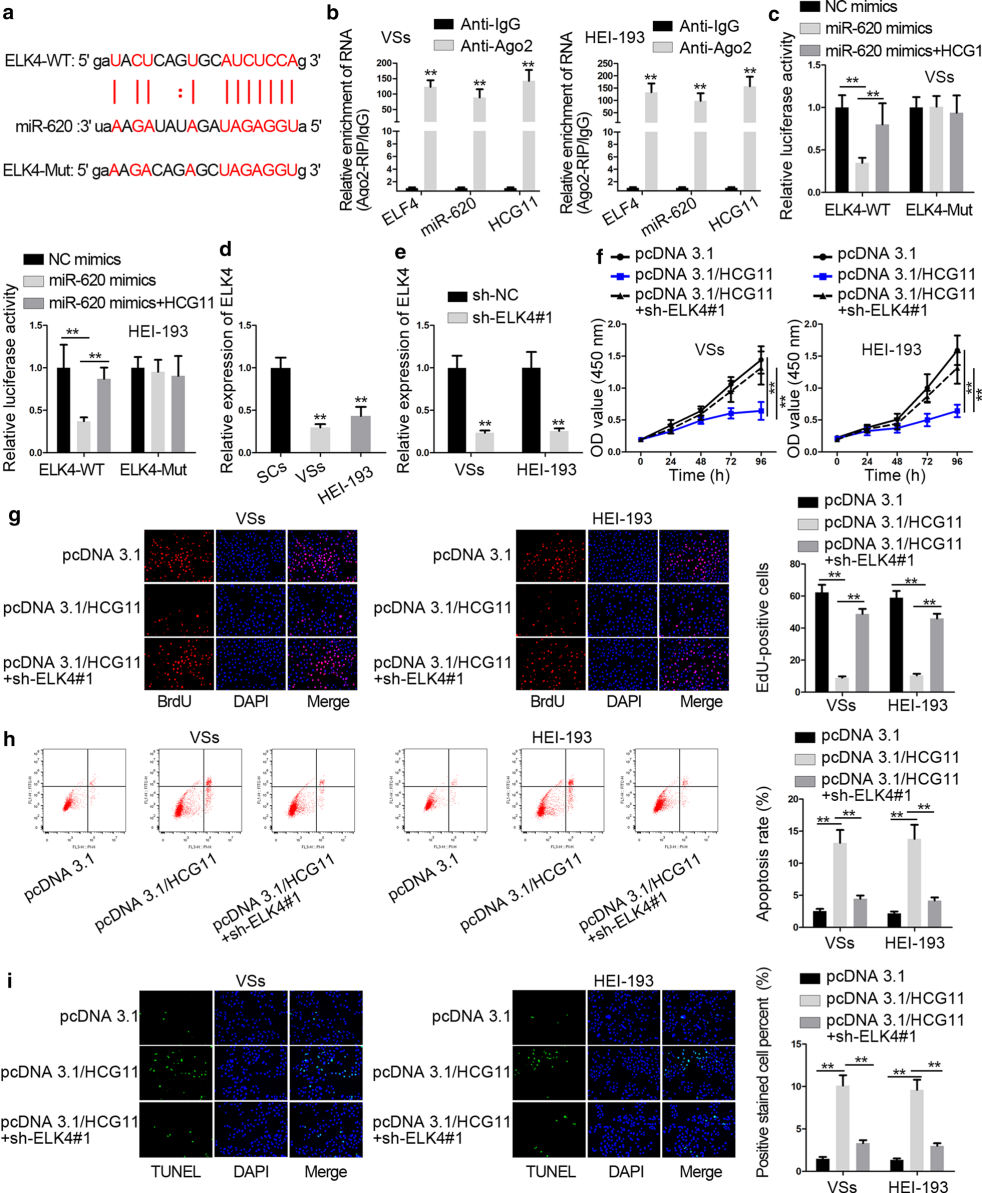
HCG11 repressed the process of VS by up-regulating ELK4 expression. **a** The binding sequences between miR-620 and ELK4 were displayed by bioinformatics. **b** RIP assays were built to corroborate ELK4, HCG11 and miR-620 coexisted in RISCs. **c** Luciferase reporter assays were performed to verify the competing relationship between ELK4 and HCG11. **d** RT-qPCR was carried out to probe the expression of ELK4 in VS cell lines. **e** Sh-ELK4#1 was transfected into cells and the expression of ELK4 was tested by RT-qPCR. **f**, **g** CCK8 and EdU assays were performed to measure cell proliferation in cells treated with pcDNA3.1/HCG11 and sh-ELK4#1. **h**, **i** Apoptosis was determined by flow cytometry analysis and TUNEL in cells transfected with pcDNA3.1/HCG11 and sh-ELK4#1. *P < 0.05, **P < 0.01

Bioinformatics predicted the binding sites between miR-620 and ELK4 (Fig. 4a). RIP assays were set up and the results depicted that HCG11, miR-620 and ELK4 were all abundant in Ago2 antibody but not in IgG antibody (Fig. 4b). Thus, they all coexisted in RNA-induced silencing RNA (RISCs). Data from the luciferase report assays showcased that the luciferase activity of cells with ELK4-WT was cut down by miR-620 mimics but there were no distinct changes in that with ELK4-Mut. More interestingly, after HCG11 was transfected into cells, the activity was restored partially (Fig. 4c). Then, the expression of ELK4 was tested in VS cells. The results presented that ELK4 expression was up-regulated in VS cell lines (Fig. 4d). Next, we studied that HCG11 could regulate the process of VS through modulating ELK4. Sh-ELK4#1 was transfected into cells and data from RT-qPCR described that ELK4 expression was lessened by sh-ELK4#1 (Fig. 4e). Then, we analyzed whether HCG11 could regulate the process of VS through modulating ELK4. VS cells received transfection with sh-ELK4#1 and pcDNA3.1/HCG11. The decreased cell proliferation ability caused by overexpression of HCG11 was rescued by knockdown of ELK4. The results were shown in CCK8 and EdU assays (Fig. 4f, g). The increased apoptosis rate induced by HCG11 up-regulation was restored by silencing ELK4 whose results illuminated in TUNEL and flow cytometry analysis (Fig. 4h, i). In a word, HCG11 constricted cell proliferation while facilitated apoptosis of VS cells via enhancing ELK4 expression.

## Discussion

Vestibular schwannoma (VS) is benign tumors caused by the inactivation of Nf2 gene (27236462). Although the malignant transformation is uncommon (27421984), VS still causes some complications to the patients, such as hearing loss, tinnitus and balance disorder. Currently, the clinical treatment remains limited owing to the lack of effective medicine (27236462). Reviewing previous research, although the diagnosis and treatment regarding eighth carnial nerve schwannoma has achieved improvement over the years, merely few biological factors able to affect tumor growth have been discussed, such as YAP, TAZ and ATEG (28430338). As the prediction of VS growth is likely to determine the therapeutic directions, it is necessary to identify factors able to project tumorigenes to acquire better prognosis.

Recently, lncRNAs are described as targets of treatment in growing studies [19, 20], which offered us inspiration to study the role of lncRNAs in regulating VS progression. HCG11 has been studied in breast cancer [21]. In this study, we examined and discovered that HCG11 was in a low expression level in VS cell lines. Overexpression of HCG11 could inhibit proliferative abilities while promote apoptosis of VS cells. This result was similar to the finding in glioma where HCG11 overexpression constricted the proliferation [10].

Then, we analyzed HCG11 in ceRNA network. Accumulating researches suggest that lncRNAs affected the development of cancers via regulating the downstream targets mRNA [22, 23]. HCG11 was reported to work as an anti-oncogene in prostate cancer by sponging miR-543 and modulating AKT/mTOR pathway [24]. In our study, first, we used FISH assay to determine the subcellular place of HCG11. Subsequently, we searched bioinformatics and selected out miRNAs which could bind with both HCG11 and ELK4. Finally miR-620 was selected as a target of HCG11. The combination between miR-620 and HCG were certified by RNA pull-down and luciferase report experiments. Moreover, we used luciferase reporter assays to verify the competing relationship between HCG11 and ELK4.

MiRNAs were described to have extremely critical functions in various cancers [25, 26]. Brazilein was described to induce apoptosis of VS by enhancing miR-133a expression [27]. MiR-620 up-regulation was delineated to forward the drug resistance in triple negative breast cancer through regulating DCTD [28]. In this study, miR-620 was examined to be high expressed in VS cell lines. Depletion of miR-620 could weaken the ability of cell proliferation while accelerated apoptosis rate of VS cell.

ELK4 was reported to have crucial roles in a host of cancers [29, 30]. In this study, we found ELK4 could regulate the transcription of HCG11 in VS cells. Furthermore, we tested the expression of ELK4 and found it was in a high level in VS cells. In addition, up-regulation of ELK4 could restore the results imposed by HCG11 overexpression in VS progression.

## Conclusion

To sum up, the data collected from our research revealed that HCG11 repressed the course of VS by cooperating miR-620/ELK4. HCG11 could be considered as a target for VS therapy in the future.

## Supplementary information

**Additional file 1: Figure S1.** The molecular axis of HCG11/miR-620/ELK4 was depicted.

## Data Availability

Not applicable.

## Abbreviations

VS: Vestibular schwannoma
lncRNAs: Long non-coding RNAs
ceRNAs: Competing endogenous RNAs
miRNAs: MicroRNAs
