# Supplementary figures and images for: HCG11 up-regulation induced by ELK4 suppressed proliferation in vestibular schwannoma by targeting miR-620/ELK4
Source: Cancer Cell Int. 2021 Jan 5;21:5. doi: 10.1186/s12935-020-01691-0 (PMC7786942; doi:10.1186/s12935-020-01691-0)

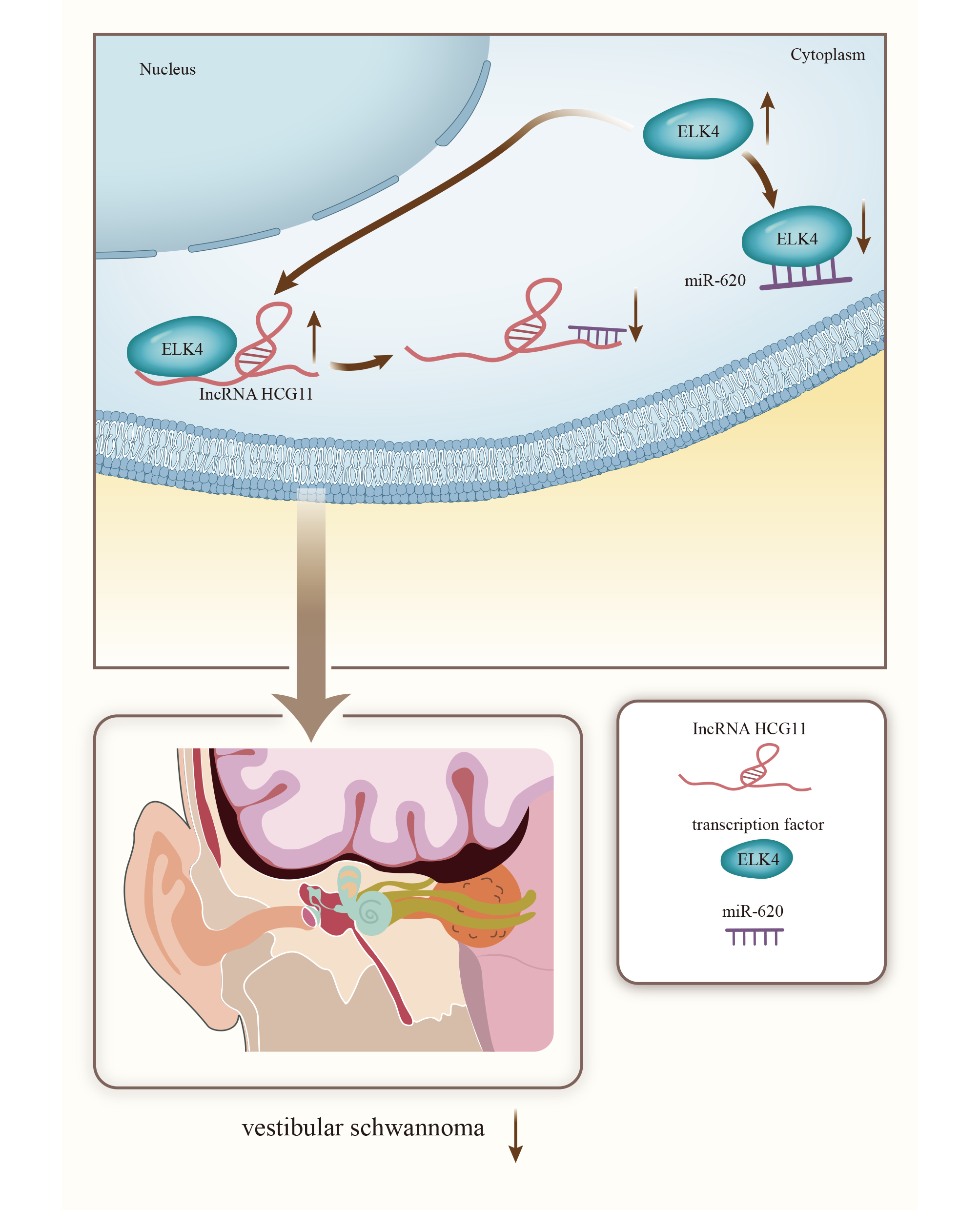

Supplement: Supplementary file 1 — Additional file 1: Figure S1. The molecular axis of HCG11/miR-620/ELK4 was depicted. [file 12935_2020_1691_MOESM1_ESM.tif]
